# The newly synthesized thiazole derivatives as potential antifungal compounds against *Candida albicans*

**DOI:** 10.1007/s00253-021-11477-7

**Published:** 2021-08-19

**Authors:** Anna Biernasiuk, Anna Berecka-Rycerz, Anna Gumieniczek, Maria Malm, Krzysztof Z. Łączkowski, Jolanta Szymańska, Anna Malm

**Affiliations:** 1 Department of Pharmaceutical Microbiology, Faculty of Pharmacy, Medical University of Lublin, Chodźki 1, 20-093 Lublin, Poland; 2 Department of Medicinal Chemistry, Faculty of Pharmacy, Medical University of Lublin, Jaczewskiego 4, 20-090 Lublin, Poland; 3 Department of Medicinal Informatics and Statistics with E-Learning Lab, Faculty of Health Sciences, Medical University of Lublin, Jaczewskiego 4, Lublin, 20-090 Poland; 4 Department of Chemical Technology and Pharmaceuticals, Faculty of Pharmacy, Collegium Medicum, Nicolaus Copernicus University, Jurasza 2, 85-089 Bydgoszcz, Poland; 5 Department of Integrated Paediatric Dentistry, Chair of Integrated Dentistry, Faculty of Medical Dentistry, Medical University of Lublin, Lubartowska 58, 20-94 Lublin, Poland

**Keywords:** Thiazole, Antifungal activity, *Candida albicans*, Mode of actions, Interactions, Lipophilicity

## Abstract

**Abstract:**

Recently, the occurrence of candidiasis has increased dramatically, especially in immunocompromised patients. Additionally, their treatment is often ineffective due to the resistance of yeasts to antimycotics. Therefore, there is a need to search for new antifungals. A series of nine newly synthesized thiazole derivatives containing the cyclopropane system, showing promising activity against *Candida* spp., has been further investigated. We decided to verify their antifungal activity towards clinical *Candida albicans* isolated from the oral cavity of patients with hematological malignancies and investigate the mode of action on fungal cell, the effect of combination with the selected antimycotics, toxicity to erythrocytes, and lipophilicity. These studies were performed by the broth microdilution method, test with sorbitol and ergosterol, checkerboard technique, erythrocyte lysis assay, and reversed phase thin-layer chromatography, respectively. All derivatives showed very strong activity (similar and even higher than nystatin) against all *C. albicans* isolates with minimal inhibitory concentration (MIC) = 0.008–7.81 µg/mL Their mechanism of action may be related to action within the fungal cell wall structure and/or within the cell membrane. The interactions between the derivatives and the selected antimycotics (nystatin, chlorhexidine, and thymol) showed additive effect only in the case of combination some of them and thymol. The erythrocyte lysis assay confirmed the low cytotoxicity of these compounds as compared to nystatin. The high lipophilicity of the derivatives was related with their high antifungal activity. The present studies confirm that the studied thiazole derivatives containing the cyclopropane system appear to be a very promising group of compounds in treatment of infections caused by *C. albicans*. However, this requires further studies in vivo.

**Key points:**

• *The newly thiazoles showed high antifungal activity and some of them — additive effect in combination with thymol.*

• *Their mode of action may be related with the influence on the structure of the fungal cell wall and/or the cell membrane.*

• *The low cytotoxicity against erythrocytes and high lipophilicity of these derivatives are their additional good properties.*

**Graphical abstract:**

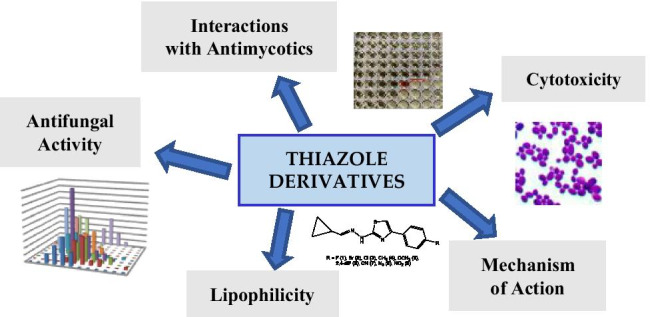

**Supplementary Information:**

The online version contains supplementary material available at 10.1007/s00253-021-11477-7.

## Introduction

Over the past years, the occurrence of systemic, life-threatening fungal infections caused by *Candida* spp. has increased dramatically, especially in patients with altered immune system (Houšť et al. 2020; Pristov and Ghannoum 2019). Besides, these yeast species may be etiological agents of superficial infections. The predominant cause of all types of candidiases is *Candida albicans* — simultaneously, the fourth most common etiological factor of hospital acquired infections. It is associated with the occurrence of mortality rates as high as 35–50% (Roemer and Krysan 2014; Turecka et al. 2018). Other emerging *Candida* species — non-*albicans Candida* spp. (NAC) like *C. glabrata*, *C. krusei*, or *C. parapsilosis *— are also serious nosocomial threats (Silva et al. 2017; Sun et al. 2015). Currently, the list of the commertially available antifungal agents, used for the treatment of infections caused by *Candida* spp., is limited to three major classes: polyenes (e.g., amphotericin B or nystatin), azoles (e.g., fluconazole or posaconazole), and echinocandins (e.g., caspofungin or micafungin) (Sharma et al. 2016; Roemer and Krysan 2014; Turecka et al. 2018). Their mechanisms of action in the fungal cell are different: polyenes bind fungal cell membrane ergosterol leading to cell lysis; azoles inhibit ergosterol biosynthesis with fungistatic activity and echinocandins block fungal (1,3)-*β*-D-glucan cell wall synthesis exhibiting fungicidal effect. Moreover, the candidiases are difficult to treat due to resistance to many antimycotics, especially azoles (Sharma et al. 2016; Silva et al. 2017; Turecka et al. 2018).

The desire to simplify traditional therapy method in which the drug mixture is used has led scientists to create hybrid drugs in which two or more pharmacophores have been combined into one structure of a new drug. This combination leads to a drug that is characterized by a higher activity, metabolic stability, ability to penetrate through various biological membranes, and reducing the microbial resistance (Viegas-Junior et al. 2007). For many years, thiazole derivatives have been widely studied due to their broad spectrum of activity, such as antimicrobial (Carradori et al. 2013; Chhabria et al. 2016; Łączkowski et al. 2016, 2017), anticancer (Gomha et al. 2016; Donarska et al. 2021; Piechowska et al. 2019), anti-*Plasmodium falciparum* (Makam et al. 2014), anti-*Trypanosoma cruzi* (de Oliveira Filho et al. 2017), anticonvulsant (Siddiqui et al. 2020), antioxidant (Salar et al. 2019) as well as anti-SARS-CoV-2 (Konno et al. 2013; Abu-Melha et al. 2020). It has been shown in literature that the thiazole scaffold is an excellent pharmacophore that can be used to develop new antimicrobial. In the last years, the cyclopropane system enjoys great interest in the drug design, and it can be found in eight of the top 200 best-selling drugs approved by the Food and Drug Administration (FDA) (Talele 2016). The cyclopropane rings are preferably used as replacements for alkyl chains, for example, the *gem*-dimethyl (Wood et al. 2006), and alkene groups (Hopkins et al. 2011), and as a substitute of phenyl ring (Abe et al. 2011) in order to increase the metabolic stability or reduce lipophilicity of the drugs. Unique properties of the cyclopropane ring are due to its planarity, low molecular weight, and conformational rigidity. Recently, series of thiazole derivatives containing the cyclopropane system, showing very high activity against *Candida* spp., has been developed. Their activity was comparable and even higher than that of nystatin (MIC = 0.015–7.81 µg/mL). The toxicity studies on cell lines showed that *Candida* spp. growth was inhibited at non-cytotoxic concentrations (Łączkowski et al. 2018).

In the present work, we verified the antifungal activity of the newly synthesized thiazole derivatives against clinical *C. albicans* strains. In addition, we examined their mechanism of antifungal action on fungal cell and the effect in combination with selected antimycotics. This work also conducted tests to assess the toxicity of these compounds to erythrocytes and their lipophilicity.

## Materials and methods

### Chemicals

**Fig. 1:**
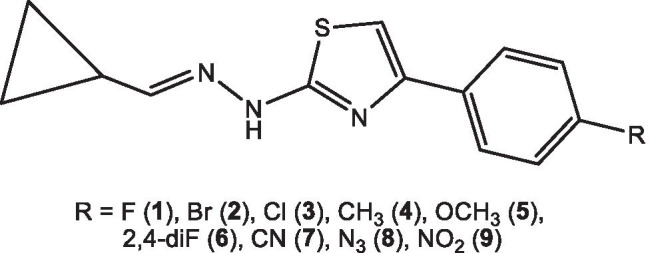
The newly synthesized thiazole derivatives with high activity against *Candida* spp

The nine newly synthesized (2-(cyclopropylmethylidene)hydrazinyl)thiazole derivatives that showed very high activity against reference and clinical isolates of *Candida* spp. in our previous studies (Łączkowski et al. 2018) were further evaluated for their biological activity. The structure of the developed thiazoles (**T1–T9**) was presented in Fig. 1, while their synthesis and all components necessary for this synthesis (Sigm-Aldrich Chemicals, St. Louis, MO, USA) were described elsewhere (Łączkowski et al. 2018).

### Microorganisms

Two references strains of yeasts from American Type Culture Collection (ATCC): *Candida albicans* ATCC 2091 and *Candida albicans* ATCC 10231 were included. Moreover, 30 clinical isolates of *C. albicans* were used. These microorganisms were isolated from oral cavity of hospitalized patients with hematological malignancies (from the collection of clinical strains deponded in Department of Pharmaceutical Microbiology of Medical University in Lublin, Poland). The Ethical Committee of the Medical University of Lublin approved the study protocol (No. KE-0254/75/2011). The isolates were identified by standard diagnostic methods: microscopic, macroscopic, and biochemical microtest, e.g., API 20 C AUX, ID 32 C, API Candida (bioMèrieux, France) on the basis of assimilation of various substrates (Biernasiuk et al. 2018; Łączkowski et al. 2018). Strains were stored as glycerol stock at −70 °C. For research purposes, fungal cultures were conducted at 35 °C for 24 h on Sabouraud agar (BioMaxima S.A., Lublin, Poland).

### Antifungal activity assay

To verify antifungal activity of the newly synthesized thiazole derivatives against clinical strains of *C. albicans*, the broth microdilution method was used according to European Committee on Antimicrobial Susceptibility Testing (EUCAST) (2003) and Clinical and Laboratory Standards Institute (CLSI) guidelines (2012). These assays were performed as described previously (Biernasiuk et al. 2018; Łączkowski et al. 2018). The samples containing 10 mg of studied compounds were dissolved in 1 mL dimethylsulphoxide (DMSO).The MIC (Minimal Inhibitory Concentration) of the compounds was examined using their two-fold dilutions in RPMI 1640 broth (Sigma-Aldrich Chemicals, St. Louis, MO, USA) with MOPS (3-(N-Morpholino)propanesulfonic acid) (Sigma-Aldrich Chemicals, St. Louis, MO, USA), prepared in 96-well polystyrene plates. Final concentrations of the thiazole derivatives ranged from 0.002 to 1000 µg/mL. All of the used isolates of *C. albicans* were first subcultured on Sabouraud agar at 37 °C for 24 h. The fungal suspensions were prepared in sterile saline (0.85% NaCl) with an optical density of McFarland standard scale 0.5 — approximately 5 × 10^5^ CFU/mL (Colony Forming Units/mL). Next, to each well containing 100 µL of RPMI 1640 broth with MOPS and the above various concentrations of tested compounds, 1 µL of the appropriate fungal suspension was added. After incubation (37 °C, 24 h), the MIC was assessed spectrophotometrically as the lowest concentration of the samples showing complete fungal growth inhibition. The DMSO, growth, and sterile controls were also carried out. Moreover, their MIC was assessed spectrophotometric as the lowest concentration of the samples showing complete fungal growth inhibition. The standard antifungal antibiotic — nystatin (Sigma-Aldrich, Chemicals, St. Louis, MO, USA) was used as positive control. In turn, the MFC (Minimal Fungicidal Concentration) defined as the lowest concentration of the compounds was required to kill a particular fungal species. MFC was determined by removing the culture using for MIC determinations from each well and spotting onto Sabouraud agar medium. Then, the plates were incubated in the appropriate conditions (as before). The lowest compound concentrations with no visible growth observed were assessed as a fungicidal concentration. All the experiments were repeated three times, and representative data are presented. In this study, very strong bioactivity was defined as a MIC < 10 µg/mL. Moreover, the MFC/MIC ratios were also calculated in order to determine fungicidal (MFC/MIC ≤ 4) or fungistatic (MFC/MIC > 4) effect of the studied thiazole derivatives (O'Donnell et al. 2010; Wiegand et al. 2008).

### Mode of action

#### Sorbitol assay

To evaluate the effect of the newly synthesized thiazole derivatives on the cell wall of *C. albicans*, the sorbitol assay was performed. The sorbitol (Sigma-Aldrich Chemicals, St. Louis, MO, USA), as an osmotic protector, was added to the culture medium in a final concentration of 0.8 M. The MIC of the tested compounds using Sabouraud Dextrose Broth (SDB) medium (BioMaxima S.A., Lublin, Poland) with and without sorbitol (control) against yeasts was determined in different lines of the same microplate. The test was performed using the microdilution technique in triplicate according to the previous guidelines (O'Donnell et al. 2010; Wiegand et al. 2008). After filling each well of the microplates with 100 µL of SDB and 100 µL of SDB supplemented with sorbitol, serial dilutions of newly thiazole derivatives and nystatin (as control) ranging from 0.004 to 1000 μg/mL were carried out. Subsequently, 10 µL of yeast suspension (10^6^ CFU/mL) was added to each well. Yeast growth and sterility control were also performed. The plates were incubated at 37 °C and read after 2 and 7 days. MICs were defined as the lowest concentrations of tested compounds capable of visually inhibiting the candidal growth. Sorbitol assay was prepared in accordance with the procedure showed by other authors (Castro and Lima 2013; Leite et al. 2014; Lima et al. 2012; de Oliveira Filho et al. 2016; Rajkowska et al. 2016; Turecka et al. 2018).

#### Ergosterol assay

To assess if the newly synthesized thiazole derivatives bind to the fungal membrane sterols, this experiment was performed. The stock solution of exogenous ergosterol (Sigma-Aldrich Chemicals, St. Louis, MO, USA) at final concentration 10 mg/mL was prepared at the time of the experiment. Ergosterol was first pulverized in the pre-sterilized porcelain mortar and dissolved in DMSO (no more than 10% of final volume) with addition 1% Tween 80 (Pol-Aura, Różnowo, Poland). The formed emulsion was then homogenized, heated to augment the solubility, and diluted with the liquid culture medium (in 89% of the final volume of liquid medium). The MIC of tested compounds against *C. albicans* was determined by using broth microdilution techniques according to the previous guidelines (O'Donnell et al., 2010; Wiegand et al. 2008) in the presence and absence of exogenous ergosterol, added to the assay medium, in different lines of the same microplate. The ergosterol was transferred to the wells in a final concentrations of 100, 200, and 400 μg/mL. After filling each well of the microplates with 100 µL of RPMI-1640 medium with and without ergosterol, serial dilutions of newly thiazole derivatives and nystatin (as positive control) ranging from 0.002 to 1000 μg/mL were carried out. Then, 10 µL of yeast suspension (10^6^ CFU/mL) was added to each well. The plates were incubated at 36 °C for 24 h and MIC was determined as the lowest concentration of tested compounds inhibiting the visible growth of *C. albicans*. Yeast growth and sterility were also controlled. Ergosterol assay was prepared according to the procedure described by other authors (Castro and Lima 2013; Leite et al. 2014; Lima et al. 2012; de Oliveira Filho et al. 2016; Rajkowska et al. 2016; Turecka et al. 2018).

### Investigation of interaction of the newly synthesized thiazole derivatives and selected antimycotics

To determine the fractional inhibitory concentrations (FICs) of the newly synthesized thiazole derivatives in combination with other antifungal substances, a checkerboard technique (according to CLSI) was used in 96-well microtiter plates. The different antimycotics for these studies were used: antifungal antibiotic — nystatin, synthetic antiseptic — chlorhexidine, and natural compound — thymol (Sigma-Aldrich Chemicals, St. Louis, MO, USA). The tested thiazole derivatives and selected antimycotics in specific concentrations (estimated at their respective MIC values) were used. These substances at various concentrations in the broth corresponding: 8 × MIC, 4 × MIC, 2 × MIC, MIC, 1/2 × MIC, 1/4 × MIC, and 1/8 × MIC (from eight times greater than their MIC to eight times lower than their MIC), were added horizontally (studied compounds) and vertically (selected antifungals) to the wells of the plate. Finally, the reference *C. albicans* inoculum was added to per each well in the plate. Growth and sterility controls were also performed. Next plates were incubated at 35 °C for 24 h. Each test was performed in triplicate (Blanco et al. 2017; Castro and Lima 2013; Turecka et al. 2018). After describing the MIC for each row, the FIC and Ʃ FIC (FIC index) were calculated as Ʃ FIC = FICA + FICB = (CA/MICA) + (CB/MICB), where MICA and MICB are the MIC_S_ of compounds A (new thiazole derivatives) and B (selected antimycotics) alone, respectively. In turn, CA and CB are the concentrations of the studied compounds: A in combinations with B and B in combinations with A, respectively. FICI – FIC index values were interpreted as follows: FICI values of ≤ 0.5 as synergy, FICI values between 0.5 and 1 as additive, FICI values between 1 and 4 as indifferent, and FICI values > 4 as antagonism (Blanco et al. 2017; Castro and Lima 2013; Turecka et al. 2018).

### Erythrocyte lysis assay

The erythrocyte lysis assay (ELA) was performed to study the toxicity of the newly synthesized thiazole derivatives on red blood cells. In the first, erythrocytes were harvested from 5.0 mL fresh sheep blood (BioMaxima S.A., Poland) by centrifugation for 10 min at 1000 × g and washed three times with 0.85% NaCl. Subsequently, 2% erythrocyte suspension was prepared in sterile phosphate buffer saline and in a volume of 100 μL was added to each well of a 96-well microtiter plate. The serial dilutions of the thiazole derivatives ranging from 0.48 to 1000 μg/mL were performed. For comparison purposes, nystatin as standard antimycotic in the same concentration range was also examined. To estimate the relative hemolytic potential of the tested compounds, the appropriate controls, i.e., 100% erythrocyte lysis using 4% Triton X-100 (Pol-Aura, Różnowo, Poland) and 0% lysis in saline solution, were used. Plates with samples were incubated for 1 hour at 37 °C, then centrifuged for 10 min at 1000 × g to separate the unlysed erythrocytes, and subsequently, the supernatant was transferred to a new plate. The absorbance was measured spectrophotometrically at 450 nm. The ELA represents an advantageous bioassay, because the lytic response can be measured photometrically by the amount of released hemoglobin. The hemolysis percentage was calculated according to the equation: % hemolysis = [(A450 of test compound treated sample-A450 of buffer treated sample)/(A450 of 4% Triton X-100 treated samples-A450 of buffer treated sample)] × 100 (Eschbach et al. 2001; Silva et al. 2017; Turecka et al. 2018; Zohra and Fawzia 2014).

### Lipophilicity

Experimental lipophilicity of the newly synthesized thiazole derivatives was determined using reversed phase thin-layer chromatography on 10 × 10 cm plates coated with C18 silica (HPTLC silica gel RP18 F_254_ from E. Merck, Darmstadt, Germany). Binary eluents were prepared by mixing appropriate volumes of water and one of the following organic modifiers in volumes increasing by 5% (50–70% of 1,4-dioxane, 50–80% of acetonitrile, 55–75% of acetone and 65–90% of methanol). All organic modifiers were of analytical grade, and they were supplied by POCH (Gliwice, Poland). The reference substances with known lipophilicity were 2-aminophenol (S1), benzocaine (S2), 2-ethyl hydroxybenzoate (S3), phenyl salicylate (S4), and 3,4-benzopyrene (S5) (Sigma-Aldrich Chemicals, St. Louis, MO, USA; Fluka, Germany and POCH, Gliwice, Poland).

The studied thiazole derivatives and the reference substances (S1–S5) were dissolved in methanol to obtain the concentration of 2.0 mg/mL. From these working solutions, volumes of 0.2 μL were spotted to the plates. The chromatograms were developed to a distance of 8 cm from the origin, in horizontal teflon chambers with an eluent distributor (Chromdes, Lublin, Poland) at constant temperature of 23 ± 1 °C. After developing of the chromatograms, the spots of the substances were localized under ultraviolet illumination at 254 nm.

On the basis of retardation coefficients (*R*_*F*_) obtained from the chromatograms, the *R*_*M*_ values for the reference substances and the thiazole derivatives were calculated using the following equation: *R*_*M*_ = log (1 − *R*_*F*_/*R*_*F*_). Then, *R*_*M*0_ values (equivalent to the retention of an analyte extrapolated to 100% water as eluent) were calculated using the following equation: *R*_*M*_ = *R*_*M*0_ − *Sφ*, where *φ* is the volume fraction of the organic modifier in the eluent and *S* is the slope of respective regression curve. The calculated *R*_*M*0_ values for the reference substances were correlated with their log *P* values found in the literature (Komsta et al. 2010) and linear calibration curves were obtained for all organic modifiers, i.e., 1,4-dioixane, acetone, acetonitrile, and methanol. Lipophilicity of the newly synthesized thiazole derivatives was calculated using their *R*_*M*0_ values on the basis of these calibration equations.

### Data analysis

All the samples were analyzed in triplicate, and representative data (mode) are presented.

## Results

### Antifungal activity

Taking into account the results presented earlier (Łączkowski et al. 2018), the newly synthesized (2-(cyclopropylmethylidene)hydrazinyl)thiazole derivatives showed very strong activity (MIC < 10 µg/mL) towards two reference *Candida albicans* ATCC 2091 and *Candida albicans* ATCC 10231 strains (with MIC = 0.015–3.91 µg/mL) (O'Donnell et al., 2010; Wiegand et al. 2008). Moreover, MFC values were also very high, in the range 0.015–15.62 µg/mL. Therefore, this study aimed to confirm the activity of these substances against 30 clinical isolates of *C. albicans* from the oral cavity of hospitalized patients with hematological malignancies. These patients are particularly vulnerable to infections, and *C. albicans* is the main etiological factor of candidiasis.

According to distribution of MIC and MFC values among clinical isolates (Figure S1) and data presented in Table 1, the assayed thiazole derivatives showed very strong antifungal effect towards these strains with MIC = 0.008–7.81 µg/mL, dependent on the compound. The values of MFC were 2-4-fold higher (MFC = 0.015–31.25 µg/mL) than MIC values. In addition, the MIC_50_ or MIC_90_ values for these compounds were calculated, defined as the minimum concentrations inhibiting the growth of 50% or 90% of all tested strains, respectively. These values were MIC_50_ = 0.12–1.95 and MIC_90_ = 0.24–3.91 µg/mL. In turn, MFC_50_ and MFC_90_ values were described as the lowest concentrations required to kill 50% or 90% of the 30 clinical *C. albicans* isolates (MFC_50_ = 0.24–7.81 and MIC_90_ = 0.48–15.62 µg/mL).

**Table 1:** The activity data (µg/mL) of the newly synthesized thiazole derivatives against 30 clinical isolates of *C. albicans* from hospitalized patients with hematological malignancies. The standard antibiotic —nystatin (NY) was used as positive control

Compounds	Range of MIC	Range of MFC	MIC_50_	MIC_90_	MFC_50_	MFC_90_
T1	0.48–3.91	0.98–7.81	0.98	1.95	1.95	7.81
T2	0.008–0.48	0.015–1.95	0.12	0.24	0.48	0.98
T3	0.015–0.98	0.06–1.95	0.12	0.48	0.48	1.95
T4	0.015–0.48	0.06–1.95	0.12	0.24	0.48	1.95
T5	0.015–1.95	0.03–7.81	0.48	0.98	1.95	3.91
T6	0.24–1.95	0.24–1.95	0.48	0.98	0.98	1.95
T7	0.48–7.81	1.95–15.62	1.95	3.91	7.81	15.62
T8	0.24–3.91	0.48–31.25	0.98	1.95	3.91	7.81
T9	0.48–3.91	0.48–3.91	0.98	1.95	1.95	3.91
NY	0.015–0.48	0.06–0.98	0.12	0.24	0.24	0.48

The highest activity showed thiazole derivatives **T2**, **T3**, and **T4** with MIC = 0.008–0.98 µg/mL, MIC_50_ = 0.12 µg/mL, and MIC_90_ = 0.24–0.48 µg/mL. The compounds **T1, T5, T6, T8**, and **T9** exhibited also strong, but slightly lower activity compared to the above mentioned derivatives (MIC = 0.015–3.91 µg/mL, MIC_50_ = 0.48–0.98 µg/mL and MIC_90_ = 0.98–1.95 µg/mL). Among the tested substances, compound **T7** showed lower activity (MIC = 0.48–7.81 µg/mL, MIC_50_ = 1.95 µg/mL, and MIC_90_ = 3.91 µg/mL). It is worth noting that the activity of the new thiazole derivatives against clinical *C. albicans* isolates was similar to nystatin, used as positive control. This activity was sometimes even stronger than nystatin, especially for compounds **T2**–**T4**. These data indicated that a series of nine newly synthesized compounds was very effective towards all studied *C. albicans* isolated from the oral cavity of patients with hematological malignancies.

Moreover, analyzing the results showed in Table 2, we observed that the studied compounds indicated mainly fungicidal activity (MFC/MIC = 1–4) against the tested clinical isolates of *C. albicans* similar to that of nystatin. The fungistatic effect (with values MFC/MIC = 8–16) was observed in a small number of *C. albicans* strains.

**Table 2:** The fungicidal/fungistatic effect of the newly synthesized thiazole derivatives against 30 clinical isolates of *C. albicans* from hospitalized patients with hematological malignancies. The standard antibiotic — nystatin (NY) was used as positive control

MFC/MIC ratio	Number (percentage) of *C. albicans* isolates									
	T1	T2	T3	T4	T5	T6	T7	T8	T9	NY
1	6 (20)	-	-	1 (3.33)	-	7 (23.33)	-	1 (3.33)	3 (10)	10 (33.33)
2	18 (60)	5 (16.66)	11 (36.66)	2 (6.67)	4 (13.33)	20 (66.67)	15 (50)	14 (46.67)	17 (56.67)	18 (60)
4	6 (20)	20 (66.67)	17 (56.67)	16 (53.33)	19 (63.33)	3 (10)	14 (46.67)	9 (30)	10 (33.33)	2 (6.67)
8	-	2 (6.67)	2 (6.67)	8 (26.67)	6 (20)	-	1 (3.33)	4 (13.33)	-	-
16	-	3 (10)	-	3 (10)	1 (3.33)	-	-	2 (6.67)	-	-

### Mode of action

The mechanism of action of the thiazole derivatives was tested in order to define whether the antifungal activity of these compounds involved a direct interaction with the cell wall structure of *C. albicans* (via testing with sorbitol) and/or with the ion permeability of the membrane of this organism (via the test with ergosterol) (Castro and Lima 2013; de Oliveira Filho et al. 2016).

#### Sorbitol assay

**Fig. 2:**
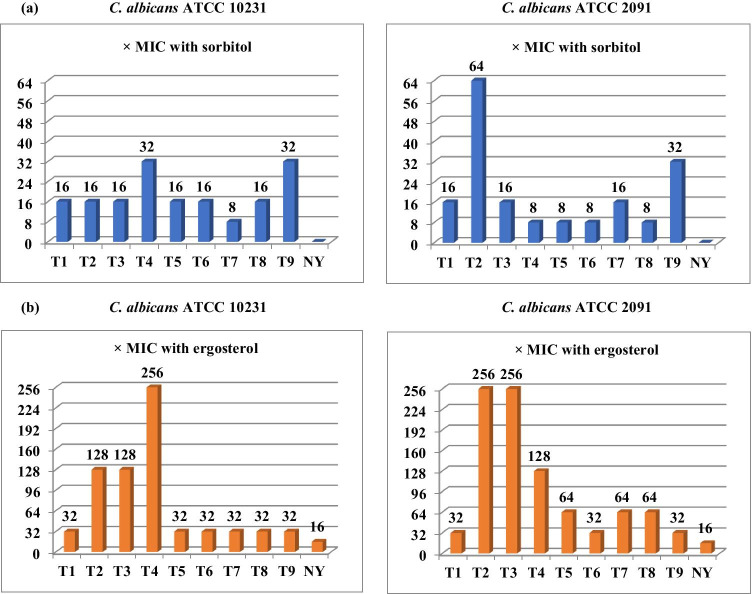
The increase in the MIC values (× MIC) of the newly synthesized thiazole derivatives in the presence of **a** sorbitol (0.8 M) and **b** ergosterol (400 µg/mL) against *C. albicans* ATCC 10231 and *C. albicans* ATCC 2091. The standard antibiotic — nystatin (NY) was used as control

**Fig. 3:**
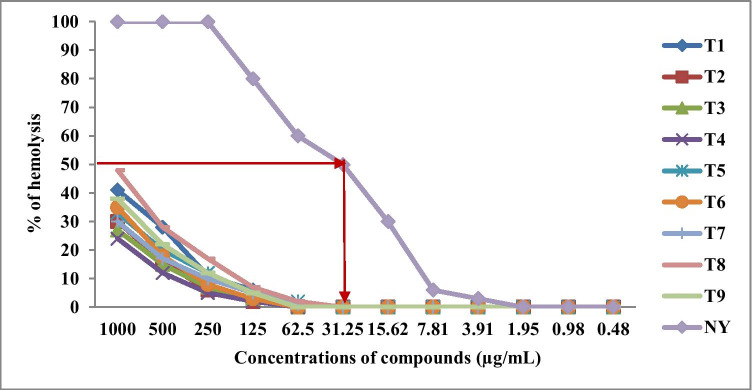
Hemolytic effect (% of hemolysis) of the newly synthesized thiazole derivatives

Sorbitol has an osmoprotectant function and is essential for fungal growth, when fungi are in the presence of drugs that act on the cell wall. It is used to stabilize fungi protoplasts, protecting their cell wall from environmental stresses, particularly osmotic changes (Leite et al. 2014; Lima et al. 2012; Rajkowska et al. 2016). As presented in Fig. 3a, the MIC values of the tested thiazole derivatives in the medium with sorbitol increased 8-32 times against *C. albicans* ATCC 10231 as compared to the cultures in the broth without sorbitol. The similar differences in MICs were also found to occur in the case of *C. albicans* ATCC 2091. The MICs of the studied compounds increased even 8–64 times in the presence of sorbitol. The MIC of nystatin used as negative control, acting at the level of fungal cell membrane, was not altered in the presence of sorbitol (Fig. 2a).

Based on the ability of sorbitol to act as a fungal cell wall osmotic protective agent, the higher MIC values observed in the medium with added sorbitol compared to the standard medium suggested that the cell wall as one of the possible cell targets for the newly synthesized thiazole derivatives because MIC values of the compounds increased in the presence of this osmotic protector (de Oliveira Filho et al. 2016; Rajkowska et al. 2016).

#### Ergosterol assay

Ergosterol and enzymes of the ergosterol biosynthetic pathway are important targets of several classes of antifungals used to treat *C. albicans* infections with a dominant position of the polyenes and the azoles (Rajkowska et al. 2016). The next step of this work was to determine if the studied thiazole derivatives act by affecting ergosterol in the fungal cell membrane.

The obtained results presented in Fig. 2b showed a clear increase in the MIC of the examined compounds in the medium with various concentrations of exogenous ergosterol (100, 200, and 400 μg/mL) compared with the ergosterol-free experiment. MIC values of these compounds and standard antibiotic for both reference *C. albicans* strains increased even 16–256 times in the presence of ergosterol. In the case of most compounds, the MIC values were 32-fold or 64-fold higher. The MIC values of studied compounds increased to the same extent at all concentrations of ergosterol tested. The highest MIC increase was found for compounds **T2**, **T3**, and **T4 **— even 256 times when ergosterol was added to the medium. Perhaps it is related with their high antifungal activity.

We included in the experiment nystatin, whose interaction with fungal cell membrane ergosterol is already known, which served as a positive control (Castro and Lima 2013; Leite et al. 2014; Lima et al. 2012; Turecka et al. 2018). The results showed a 16-fold increase in the nystatin MIC values after the addition of ergosterol, independent on its concentration. These data are consistent with previous studies of Castro and Lima (2013), in which MIC value of this antibiotic against *C. albicans* also increased 16-times in the presence of sterol. The obtained data suggest that the synthesized compounds appear to bind to the ergosterol in the membrane, which increases ion permeability and ultimately results in cell death (Castro and Lima 2013; Turecka et al. 2018). Sorbitol and ergosterol assay data suggest that the newly thiazole derivatives may act not only at the level of fungal cell wall but also at the level of fungal cell membrane.

### Investigation of interaction of newly synthesized thiazole derivatives and selected antimycotics

In the next stage of research, combination of newly synthesized thiazole derivatives with some antifungal drugs (nystatin), synthetic antiseptics (chlorhexidine) or natural substances (thymol) was also assayed for their effect on the growth of reference *C. albicans* strains in order to their define possible interactions. MICs of compounds alone, as well as MICs of combinations which exhibited inhibitory effects, were used to calculate fractional inhibitory concentrations (FICs) and Ʃ FIC (FICI – FIC index) values. To determine the interactions between the tested compounds and the chosen antifungal substances, FIC values were calculated according to the formula given by Blanco et al. (2017).

Our results showed that in combination with the nystatin or chlorhexidine, as presented in Table S1 and Table S2, was observed no growth reduction of MIC values of all studied compounds against both reference *C. albicans* strains. Their values of MICs alone and MICs in combination were the same (FIC = 1) or differed 2 times (FIC = 2) — MICs of compounds in combination were 2-fold higher than their MICs alone. The combinations of the studied compounds with nystatin (Ʃ FIC = 2–4) and chlorhexidine (Ʃ FIC = 2–3) were found to be noninterfering (FICI values between 1 and 4 were considered as indifferent).

Moreover, these results showed additive effect of compounds **T2** and **T5** in combination with thymol (Ʃ FIC = 1) against both reference yeast strains. In turn, in the case of *C. albicans* ATCC 10231, additivity was also indicated for thiazole derivatives **T3** and **T7**. The FIC values in all combinations were 0.5 towards both strains, which means a 2-fold decrease MIC values of compounds in combination. For the other derivatives, no interactions was showed (Table S3). These results indicated a good effect of combination of some thiazoles with natural compound—thymol.

### Erythrocyte lysis assay

In the present studies, the erythrocyte model (erythrocyte lysis assay — ELA) was used to assess the effect of the studied compounds on cell membrane. Hemolysis is due to red blood cell destruction which resulted from lysis of membrane lipid bilayer, and it relates with concentration and potency of the studied agents (Han et al. 2016; Zohra and Fawzia 2014). Nystatin was used in this experiment as positive control acting on the level of cell membrane.

As presented in Fig. 3, the highest concentrations of the studied compounds not exerting any hemolytic effects were in the range 31.25–62.5 µg/mL, while that of nystatin was 1.95 µg/mL. The concentration of nystatin causing 50% of hemolysis was 31.25 µg/mL. In turn, in the case of all newly developed thiazole derivatives, these concentration was above 1000 µg/mL. Data obtained using ELA confirm that antifungal effect of the newly synthesized compounds against clinical isolates of *C. albicans* (MIC = 0.008–7.81 µg/mL) was observed at their non-cytotoxic concentrations.

### Lipophilicity determination

It is well known that chromatographic methods allow fast and reproducible determination of experimental lipophilicity, especially for a wide range of new synthesized compounds. In the present study, the standardization procedure with five reference substances (S1–S5) covering the large range of lipophilicity (log *P* in the range 0.68–6.04) was used. As a result, lipophilicity of S1–S5 was highly correlated with their *R*_*M*0_ values. The coefficients of determination (*r*^2^) for respective regression equations were above 0.93 for three organic modifiers, i.e., acetone, acetonitrile, and methanol. Moreover, the *r*^2^ values were rather high (above 0.8) for the fourth organic modifier, i.e., 1,4-dioxane. Detailed data are available in Table 3.

**Table 3:** The log *P* and *R*_*M*0_ values of the reference substances (S1–S5)

Substance	log *P*	*R*_*M*0_	*S*	*r*^2^	*φ*
*1,4-Dioxane–water*					
S1	0.62	1.68	− 0.03	0.9204	53.63
S2	1.86	1.38	− 0.03	0.9242	51.34
S3	2.47	1.69	− 0.03	0.9917	53.48
S4	3.80	3.46	− 0.05	0.8684	73.97
S5	6.04	4.14	− 0.05	0.9695	79.58
*Acetone–water*					
S1	0.62	1.64	− 0.03	0.9452	60.29
S2	1.86	1.75	− 0.03	0.9720	58.40
S3	2.47	1.78	− 0.03	0.9380	58.77
S4	3.80	3.61	− 0.05	0.9956	74.96
S5	6.04	4.78	− 0.06	0.9704	82.68
*Acetonitrile–water*					
S1	0.62	1.24	− 0.01	0.9747	65.83
S2	1.86	1.73	− 0.03	0.9902	55.78
S3	2.47	1.96	− 0.04	0.9733	53.50
S4	3.80	2.64	− 0.03	0.9899	77.28
S5	6.04	4.26	− 0.05	0.9409	95.22
*Methanol–water*					
S1	0.62	2.58	− 0.03	0.9880	82.69
S2	1.86	2.18	− 0.03	0.9749	65.83
S3	2.47	2.35	− 0.03	0.9703	68.69
S4	3.80	3.61	− 0.04	0.9917	87.71
S5	6.04	5.30	− 0.05	0.9964	103.80

In addition, correlations between log *P*_EXP_ and *R*_*M*0_ of the reference substances were sufficiently high. The *r*^2^ values for respective regression equations were above 0.83 for all organic modifiers, i.e., 1,4-dioxane, acetone, acetonitrile, and methanol:

$$log PEXP=1.5180RM0-0.7915,r2=0.8332(\mathrm{1,4}-dioxane)$$

$$log PEXP=1.3876RM0-0.8049,r2=0.9036(acetone)$$

$$log PEXP=1.7467RM0-1.1756,r2=0.9787(acetonitrile)$$

$$log PEXP=1.4576RM0-1.7119,r2=0.8359(methanol)$$

Similar to the reference compounds, correlations between the *R*_*M*0_ and *R*_*F*_ values for newly synthesized thiazole derivatives were sufficiently high (*r*^2^ > 0.91) for all eluents, providing accuracy for further lipophilicity determination. These data are presented in Table 4.

**Table 4:** The *R*_*M*0_ values of the of the newly synthesized thiazole derivatives

Compound	*R*_*M*0_	*S*	*r*^2^	*φ*
*1,4-Dioksane–water*				
T1	3.99	− 0.06	0.9290	69.58
T2	3.54	− 0.05	0.8517	73.71
T3	3.46	− 0.05	0.8453	73.39
T4	3.18	− 0.04	0.8154	72.23
T5	2.83	− 0.04	0.7888	67.98
T6	3.77	− 0.05	0.8579	73.39
T7	2.93	− 0.04	0.8098	67.29
T8	3.55	− 0.05	0.8255	73.88
T9	3.14	− 0.05	0.7955	69.56
*Acetone–water*				
T1	3.12	− 0.04	0.9669	71.91
T2	3.62	− 0.05	0.9738	75.46
T3	3.51	− 0.05	0.9831	74.66
T4	2.93	− 0.04	0.9305	73.30
T5	2.43	− 0.03	0.9147	71.08
T6	3.67	− 0.05	0.9906	74.88
T7	2.46	− 0.03	0.9618	72.29
T8	3.69	− 0.05	0.9650	75.08
T9	3.79	− 0.05	0.9901	72.33
*Acetonitrile–water*				
T1	2.62	− 0.03	0.9919	75.60
T2	3.04	− 0.04	0.9983	80.64
T3	2.79	− 0.03	0.9945	80.59
T4	2.60	− 0.03	0.9875	80.07
T5	2.48	− 0.03	0.9913	75.37
T6	2.78	− 0.04	0.9869	79.53
T7	2.51	− 0.03	0.9863	72.34
T8	2.81	− 0.04	0.9804	79.35
T9	2.67	− 0.04	0.9950	74.94
*Methanol–water*				
T1	4.03	− 0.05	0.9911	85.38
T2	4.54	− 0.05	0.9459	90.87
T3	4.89	− 0.05	0.9883	88.71
T4	4.19	− 0.05	0.9939	89.21
T5	4.12	− 0.05	0.9933	86.75
T6	4.94	− 0.06	0.9842	88.17
T7	3.77	− 0.05	0.9953	82.03
T8	4.83	− 0.05	0.9689	88.88
T9	4.21	− 0.05	0.9920	85.22

As far as the experimental lipophilicity of the synthesized compounds was concerned, the highest log *P* values were calculated for 1,4-dioxane-water and methanol-water binary mixtures. In the literature, these two organic modifiers were recommended as the most suitable solvents for such estimations (Rutkowska et al. 2013). For all organic modifiers used, i.e., 1,4-dioxane, acetone, acetonitrile, and methanol, the high log *P* values was obtained for **T2** derivative containing brome atom as the substituent, while the lowest lipophilicity values were calculated for compounds **T5** and **T7** with methoxy and cyano groups. The mean log *P* values of the derivatives lied in the range 3.38–4.49 (Table 5). Generally, it is known that high lipophilic nature of the compounds facilitates reaching their site of action inside the fungal cells, which was confirmed by the lipophilicity data of the thiazole derivatives.

**Table 5:** Log *P*_EXP_ values of the newly synthesized thiazole derivatives calculated using the standardization method

Compounds	log *P*_1,4-dioxane_	log *P*_acetone_	log *P*_acetonitrile_	log *P*_methanol_
T1	5.27	3.53	3.40	4.15
T2	4.58	4.22	4.13	4.91
T3	4.47	4.06	3.70	5.42
T4	4.03	3.26	3.36	4.40
T5	3.50	2.57	3.16	4.30
T6	4.93	4.29	3.69	5.49
T7	3.66	2.61	3.20	3.79
T8	4.59	4.32	3.74	5.33
T9	3.98	4.45	3.49	4.43

## Discussion

The incidence of fungal infections has been steadily increasing in recent years (Karpiński 2020). *Candida* species, especially *C. albicans*, is a common opportunistic fungal pathogen that may cause nosocomial fungal infections, mainly in immunocompromised patients [Gong et al., 2019]. Oropharyngeal candidiasis are the most common fungal infections in these persons [Terças et al. 2017]. Although the antifungal drugs used in clinical treatment appear to be diverse, the list of antimycotics is very limited. Moreover, only few classes of antifungals are currently available to treat infections caused by *Candida* spp. It is worth adding that many of them have been extensively used and led to the development of antifungal resistance and it brings a great challenge in clinical practice. Therefore, there is a need to search for new antifungals (Sharma et al. 2016; Roemer and Krysan 2014; Turecka et al. 2018; Gong et al. 2019). In view of the high antifungal activity of group of compounds containing the cyclopropane system and their very low toxicity to human cells, we have decided to continue our research on the newly thiazole derivatives (Łączkowski et al. 2018). In the future, there is a possibility of using them as a new type of antifungal drugs. As a result of studies, we have indicated that novel (2-(cyclopropylmethylidene)hydrazinyl)thiazole showed very strong antifungal activity with MIC < 10 µg/mL (O'Donnell et al., 2010; Wiegand et al. 2008). The values of MIC ranged from 0.008 to 7.81 µg/mL towards, not only reference but also clinical isolates of *C. albicans* from patients with hematological malignancies. These persons are particularly vulnerable to infections, including endogenous candidiasis, that are frequent complications and cause of their morbidity and mortality (Nucci and Anaissie, 2018). Antimicrobial effect of new thiazoles against clinical yeasts was similar to nystatin (used as a control drug), and sometimes even higher than it (especially for compounds **T2**–**T4**). In the case of these derivatives, MICs were even below 1 µg/mL (MIC = 0.008–0.98 µg/mL). In turn, MICs below 4 µg/mL were showed for the remaining compounds: **T1**, **T5**, **T6**, **T8**, and **T9** (MIC = 0.015–3.91 µg/mL). Only compound **T7** indicated slightly lower activity (MIC = 7.81 µg/mL). Our data confirmed that a series of nine new compounds was very effective towards all studied *C. albicans* isolates and these strains were highly sensitive to them.

The results of some research, carried out by other authors (Omran et al. 2018; Eksi et al. 2013; Dagi et al. 2016), showed that conventional antifungal drugs have high and similar activity to our compounds. Namely, strains of *C. albicans* isolated from oropharyngeal infections were susceptible to caspofungin (with MIC = 0.008–1 µg/mL), amphotericin B (MIC = 0.008–0.25 µg/mL), and azoles: voriconazole, itraconazole, ketokonazole, and posaconazole (MIC = 0.016–2 µg/mL). However, resistance to fluconazole was also observed at 15.5% *C. albicans* isolates (MIC = 0.016–16 µg/mL) (Omran et al. 2018). The data of Eksi et al. (2013) presented also the same effect of amphoterin B, voriconazole (MIC = 0.03–0.25 µg/mL), and caspofungin (MIC = 0.015–0.25 µg/mL) against *C. albicans* isolates from the hospitalized patients. The values of MICs of fluconazole were lower (0.25–32 µg/mL). The similar activity towards *C. albicans* isolates from bloodstream showed Dagi et al. (2016) for amphotericin B (MIC = 0.12–1 µg/mL), azole: fluconazole (MIC = 0.12–2 µg/mL), voriconazole (MIC = ≤ 0.015–0.06 µg/mL), posaconazole (MIC = ≤ 0.015–0.12 µg/mL), and echinocandins: caspofungin (MIC = ≤ 0.008–0.12 µg/mL) and anidulafungin (MIC = 0.015–0.12 µg/mL). Many authors reported different results regarding susceptibility of *C. albicans* to antifungal agents: high activity of polyenes and echinocandins and decreased susceptibility to azoles. In some studies, resistance rates of *C. albicans* to antimycotics was varied in the range of 3.3–6.7% for polyene and 0.2–0.5% for echinocandins [Hedayati et al. 2019; Badiee et al. 2017; Dagi et al. 2016]. In turn, a significant resistance of 56.5–64.5% to azoles was found. The high use of antimycotics including fluconazole was showed in some hospitals which reported a higher population of patients with hematological malignancies. It has led to azole resistance in a high percentage of *C. albicans* strains which were frequently observed in oropharyngeal candidiasis of these patients (Zaidi et al. 2018).The antifungal agents target three cellular components of fungi. Azoles inhibit the synthesis of ergosterol in the endoplasmic reticulum of the fungal cell. Polyenes bind to ergosterol in the fungal membrane causing disruption of membrane structure and function. Flucytosine is converted within the fungal cell to 5-fluorouracil, which inhibits DNA synthesis [Terças et al. 2017]. The mechanism of action (a direct interaction with the cell wall structure and/or with the ion permeability of the membrane of *C. albicans*) of the new compounds was also investigated. It is important to know the mode of action of a compound, because this information can be used to increase the effectiveness of the substance and to decrease the selection of resistant strains (Castro and Lima 2013; de Oliveira Filho et al. 2016; Lima et al. 2012). In one of our research studies, sorbitol was used. It is a known osmostabilizer that protects the cell wall from lysis caused by antifungal agents. Sorbitol maintains proper osmotic pressure, thereby providing a suitable environment for the cell wall biosynthesis pathway (Turecka et al. 2018). We also determined whether the tested thiazole derivatives had an effect on ergosterol in the fungal cell membrane. In the case of the most active compounds **T2**, **T3**, and **T4** was found the highest (even 128–256 times) increase in MIC values when ergosterol was added to the medium. Moreover, an 8–64-fold increase in their MIC values was showed in the presence of sorbitol. Perhaps it is related with their high antifungal activity. The results indicated that the mechanism of action of studied compounds may be related to their interaction within the cell wall structure of *C. albicans* and within their cell membrane. It is worth noting that the high activity was obtained for **T2, T3**, and **T4** derivatives containing brome atom, chlor atom, and methyl group as the substituent, respectively. Some of them, mainly **T2** derivative, had the high lipophilicity, while the lowest lipophilicity values were calculated for compounds **T5** and **T7** with methoxy and cyano groups. The high lipophilic nature of the compounds facilitates reaching their site of action inside the fungal cells and increases this activity. Additionally, combination of newly synthesized thiazole derivatives with different antifungal compounds was also assayed for their effect on the growth of reference *C. albicans* strains in order to their define possible interactions. Some compounds (**T2**, **T3**, **T5**, and **T7**) showed additive effect in combination with the natural substance — thymol. In the case of nystatin (antifungal antibiotic) and chlorhexidine (synthetic antiseptic), no interaction was showed. The combined application of antifungals provides for a reduction of their doses, improves their efficacy, or reduces toxicity. As reported in our previous paper (Łączkowski et al. 2018), the highest possible non-cytotoxic concentration of the newly synthesized thiazole derivatives determined on the cell line model (the mouse L929 fibroblast and the African green monkey kidney — Vero cells) was found to be 7.81–31.25 µg/mL, dependent on the compound. In the present studies, the erythrocytes were used to assess the influence of the tested compounds on their cell membrane. The red blood cell lysis was related with these derivatives in the range of concentrations from 31.25 to 62.5 µg/mL. The erythrocyte lysis assay confirmed also the low cytotoxicity of tested compounds. The *Candida* spp. growth was inhibited at their non-cytotoxic concentrations. In conclusion, all reference strains and *C. albicans* isolates from oral cavity of hospitalized patients with hematological malignancies were susceptible to the novel (2-(cyclopropylmethylidene)hydrazinyl)thiazole at MIC < 10 µg/mL. These compounds had a strong anticandidal effect (similar to conventional antimycotics) at non-cytotoxic concentrations. Their mechanism of activity may be associated with the action within the fungal cell wall structure and/or within the cell membrane. Moreover, the high lipophilicity of the derivatives may be related with their high antifungal activity. Therefore, in the field of antifungals, searching new the studied thiazole derivatives appears to be a very promising group of compounds, which may be used, in the future, in the treatment of candidiasis.

## Supplementary Information

Below is the link to the electronic supplementary material.

Supplementary file1 (PDF 358 kb)

## Data Availability

Data and material for this article are available upon request.

## References

[CR1] Abe H, Kikuchi S, Hayakawa K, Iida T, Nagahashi N, Maeda K, Sakamoto J, Matsumoto N, Miura T, Matsumura K, Seki N, Inaba T, Kawasaki H, Yamaguchi T, Kakefuda R, Nanayama T, Kurachi H, Hori Y, Yoshida T, Kakegawa J, Watanabe Y, Gilmartin AG, Richter MC, Moss KG, Laquerre SC (2011). Discovery of a highly potent and selective MEK inhibitor: GSK1120212 (JTP-74057 DMSO Solvate). ACS Med Chem Lett.

[CR2] Abu-Melha S, Edrees MM, Riyadh SM, Abdelaziz MR, Elfiky AA, Gomha SM (2020). Clean grinding technique: A facile synthesis and *in silico* antiviral activity of hydrazones, pyrazoles, and pyrazines bearing thiazole moiety against SARS-CoV-2 Main Protease (Mpro). Molecules.

[CR3] Badiee P, Badali H, Boekhout T, Diba K, Moghadam AG, Hossaini Nasab A, Jafarian H, Mohammadi R, Mirhendi H, Najafzadeh MJ, Shamsizadeh A, Soltani J (2017) Antifungal susceptibility testing of *Candida* species isolated from the immunocompromised patients admitted to ten university hospitals in Iran: comparison of colonizing and infecting isolates. BMC Infect Dis 21; 17(1): 727–735. 10.1186/s12879-017-2825-7 10.1186/s12879-017-2825-7 PMC5697407 29157206

[CR4] Biernasiuk A, Szymańska J, Kustra A, Korona-Głowniak I, Malm A (2018). *In vitro* susceptibility of oral *Candida albicans* isolates to chlorhexidine. Acta Pol Pharm.

[CR5] Blanco AR, Nostro A, D'Angelo V, D’Arrigo M, Mazzone MG, Marino A (2017). Efficacy of a fixed combination of tetracycline, chloramphenicol, and colistimethate sodium for treatment of *Candida albicans* keratitis. Invest Ophthalmol vis Sci.

[CR6] Carradori S, Secci D, Bolasco A, Rivanera D, Mari E, Zicari A, Lotti LV, Bizzarri B (2013). Synthesis and cytotoxicity of novel (thiazol-2-yl)hydrazine derivatives as promising anti-*Candida* agents. Eur J Med Chem.

[CR7] Castro RD, Lima EO (2013). Anti-*Candida* activity and chemical composition of *Cinnamomum zeylanicum blume* essential oil. Braz Arch Biol Technol.

[CR8] Chhabria MT, Patel S, Modi P, Brahmkshatriya PS (2016). Thiazole: a review on chemistry, synthesis and therapeutic importance of its derivatives. Curr Top Med Chem.

[CR9] Clinical and Laboratory Standards Institute. Reference method for broth dilution antifungal susceptibility testing of yeasts. M27-S4 (2012). Clinical and Laboratory Standards Institute, Wayne, PA, USA.

[CR10] Dagi HT, Findik D, Senkeles C, Arslan U (2016). Identification and antifungal susceptibility of *Candida* species isolated from bloodstream infections in Konya. Turkey Ann Clin Microbiol Antimicrob.

[CR11] Donarska B, Świtalska M, Płaziński W, Wietrzyk J, Łączkowski KZ (2021). Effect of the dichloro-substitution on antiproliferative activity of phthalimide-thiazole derivatives: rational design, synthesis, elastase, caspase 3/7, and EGFR tyrosine kinase activity and molecular modeling study. Bioorg Chem.

[CR12] Eschbach E, Scharsack JP, John U, Medlin LK (2001). Improved erythrocyte lysis assay in microtitre plates for sensitive detection and efficient measurement of haemolytic compounds from ichthyotoxic algae. J Appl Toxicol.

[CR13] European Committee for Antimicrobial Susceptibility Testing (EUCAST) determination of minimum inhibitory concentrations (MICs) of antibacterial agents by broth dilution. EUCAST discussion document E. Dis 5.1 (2003) Clin Microbiol Infect 9:1–7.

[CR14] Eksi F, Gayyurhan ED, Balci I (2013). *In vitro* susceptibility of *Candida* species to four antifungal agents assessed by the reference broth microdilution method. Sci World J.

[CR15] Gomha S, Edrees M, Altalbawy F (2016). Synthesis and characterization of some new bis-pyrazolyl-thiazoles incorporating the thiophene moiety as potent anti-tumor agents. Int J Mol Sci.

[CR16] Gong Y, Liu W, Huang X, Hao L, Li Y, Sun S (2019). Antifungal activity and potential mechanism of N-Butylphthalide alone and in combination with fluconazole against *Candida albicans*. Front Microbiol.

[CR17] Han J, Jyoti MA, Song HY, Jang WS (2016). Antifungal activity and action mechanism of histatin 5-halocidin hybrid peptides against *Candida* ssp. PLoS ONE.

[CR18] Hedayati MT, Tavakoli M, Zakavi F, Shokohi T, Mofarrah R, Ansari S, Armaki MT (2019). *In vitro* antifungal susceptibility of *Candida* species isolated from diabetic patients. Rev Soc Bras Med Trop.

[CR19] Hopkins CD, Schmitz JC, Chu E, Wipf P (2011). Total synthesis of (-)-CP_2_-disorazole C_1_. Org Lett.

[CR20] Houšť J, Spížek J, Havlíček V (2020). Antifungal Drugs Metabolites.

[CR21] Karpiński TM (2020) Essential oils of Lamiaceae family plants as antifungals. Biomolecules 10, 103; 10.3390/biom10010103 10.3390/biom10010103 PMC7023020 31936168

[CR22] Komsta Ł, Skibiński R, Berecka A, Gumieniczek A, Radkiewicz B, Radoń M (2010). Revisiting thin-layer chromatography as a lipophilicity determination tool - a comparative study on several techniques with a model solute set. J Pharm Biomed Anal.

[CR23] Konno S, Thanigaimalai P, Yamamoto T, Nakada K, Kakiuchi R, Takayama K, Yamazaki Y, Yakushiji F, Akaji K, Kiso Y, Kawasaki Y, Chen S-E, Freire E, Hayashi Y (2013). Design and synthesis of new tripeptide-type SARS-CoV 3CL protease inhibitors containing an electrophilic arylketone moiety. Bioorg Med Chem.

[CR24] Leite MC, Bezerra AP, de Sousa JP, Guerra FQ, Lima E de O (2014) Evaluation of antifungal activity and mechanism of action of citral against *Candida albicans*. Evid Based Complement Alternat Med 378280. 10.1155/2014/378280 10.1155/2014/378280 PMC4163309 25250053

[CR25] Lima IO, de Medeiros Nóbrega F, de Oliveira WA,. Lima E de O, Menezes EA, Cunha FA, Diniz M, de FM (2012) Anti-*Candida albicans* effectiveness of citral and investigation of mode of action. Pharm Biol 50:1536-1541. 10.3109/13880209.2012.694893 10.3109/13880209.2012.694893 23116193

[CR26] Łączkowski KZ, Biernasiuk A, Baranowska-Łączkowska A, Zielińska S, Sałat K, Furgała A, Misiura K, Malm A (2016). Synthesis, antimicrobial and anticonvulsant screening of small library of tetrahydro-2H-thiopyran-4-yl based thiazoles and selenazoles. J Enzyme Inhib Med Chem.

[CR27] Łączkowski KZ, Konklewska N, Biernasiuk A, Malm A, Sałat K, Furgała A, Dzitko K, Bekier A, Baranowska-Łączkowska A, Paneth A (2018). Thiazoles with cyclopropyl fragment as antifungal, anticonvulsant, and anti-*Toxoplasma gondii* agents: synthesis, toxicity evaluation, and molecular docking study. Med Chem Res.

[CR28] Łączkowski KZ, Landowska K, Biernasiuk A, Sałat K, Furgała A, Plech T, Malm A (2017). Synthesis, biological evaluation and molecular docking studies of novel quinuclidinone derivatives as potential antimicrobial and anticonvulsant agents. Med Chem Res.

[CR29] Makam P, Thakur PK, Kannan T (2014). *In vitro* and *in silico* antimalarial activity of 2-(2-hydrazinyl)thiazole derivatives. Eur J Pharm Sci.

[CR30] Nucci M, Anaissie EJ (2018) Prevention of infections in patients with hematological malignancies. in: Wiernik P, Dutcher J, Gertz M (eds) Neoplastic diseases of the blood. Springer, Cham. 10.1007/978-3-319-64263-5_49

[CR31] O'Donnell F, Smyth TJ, Ramachandran VT, Smyth WF (2010). A study of the antimicrobial activity of selected synthetic and naturally occurring quinolones. Int J Antimicrob Agents.

[CR32] de Oliveira Filho AA, de Oliveira HMBF, de Sousa JP, Meireles D, de Azevedo Maia GL, Filho JMB, Lima EO (2016). *In vitro* anti-*Candida* activity and mechanism of action of the flavonoid isolated from *Praxelis clematidea* against *Candida albicans* species. J App Pharm Sci.

[CR33] de Oliveira Filho GB, Cardoso MVDO, Espíndola JWP, Oliveira de Silva DA, Ferreira RS, Coelho PL, Anjos PSD, Santos EDS, Meira CS, Moreira DRM, Soares MBP, Leite ACL (2017). Structural design, synthesis and pharmacological evaluation of thiazoles against *Trypanosoma cruzi*. Eur J Med Chem.

[CR34] Omran MS, Dastjerdi RM, Zuashkiani M, Moqarabzadeh V, Taghizadeh-Armaki M (2018). *In vitro* antifungal susceptibility of *Candida* species isolated from iranian patients with denture stomatitis. Biomed Res Int.

[CR35] Piechowska K, Świtalska M, Cytarska J, Jaroch K, Łuczykowski K, Chałupka J, Wietrzyk J, Misiura K, Bojko B, Kruszewski S, Łączkowski KZ (2019). Discovery of tropinone-thiazole derivatives as potent caspase 3/7 activators, and noncompetitive tyrosinase inhibitors with high antiproliferative activity: Rational design, one-pot tricomponent synthesis, and lipophilicity determination. Eur J Med Chem.

[CR36] Pristov KE, Ghannoum MA (2019). Resistance of *Candida* to azoles and echinocandins worldwide. Clin Microbiol Infect.

[CR37] Rajkowska K, Nowak A, Kunicka-Styczyńska A, Siadur A (2016). Biological effects of various chemically characterized essential oils: investigation of the mode of action against *Candida albicans* and HeLa cells. RSC Adv.

[CR38] Roemer T, Krysan DJ (2014) Antifungal drug development: challenges, unmet clinical needs, and new approaches. Cold Spring Harb Perspect Med 4 pii:a019703. doi: 10.1101/cshperspect.a019703 10.1101/cshperspect.a019703 PMC3996373 24789878

[CR39] Rutkowska E, Pająk K, Jóźwiak K (2013). Lipophilicity – methods of determination and its role in medicinal chemistry. Acta Pol Pharm - Drug Res.

[CR40] Salar U, Khan KM, Chigurupati S, Syed S, Vijayabalan S, Wadood A, Riaz M, Ghufran M, Perveen S (2019). New hybrid scaffolds based on hydrazinyl thiazole substituted coumarin; As novel leads of dual potential; in vitro α-amylase inhibitory and antioxidant (DPPH and ABTS radical scavenging) activities. Med Chem.

[CR41] Sharma Y, Khan LA, Manzoor N (2016). Anti-*Candida* activity of geraniol involves disruption of cell membrane integrity and function. J Mycol Med.

[CR42] Silva S, Rodrigues CF, Araújo D, Rodrigues ME, Henriques M (2017). *Candida* species biofilms’ antifungal resistance. J Fungi.

[CR43] Siddiqui AA, Partap S, Khisal S, Yar MS, Mishra R (2020). Synthesis, anti-convulsant activity and molecular docking study of novel thiazole pyridazinone hybrid analogues. Bioorg Chem.

[CR44] Sun L, Liao K, Wang D (2015). Effects of magnolol and honokiol on adhesion, yeast-hyphal transition, and formation of biofilm by *Candida albicans*. PLoS ONE.

[CR45] Talele TT (2016). The “cyclopropyl fragment” is a versatile player that frequently appears in preclinical/clinical drug molecules. J Med Chem.

[CR46] Terças AL, Marques SG, Moffa EB, Alves MB, de Azevedo CM, Siqueira WL, Monteiro CA (2017). Antifungal drug susceptibility of *Candida* species isolated from HIV-positive patients recruited at a public hospital in São Luís, Maranhão. Brazil Front Microbiol.

[CR47] Turecka K, Chylewska A, Kawiak A, Waleron KF (2018). Antifungal activity and mechanism of action of the Co(III) coordination complexes with diamine chelate ligands against reference and clinical strains of *Candida* spp. Front Microbiol.

[CR48] Viegas-Junior C, Danuello A, da Silva BV, Barreiro EJ, Manssour Fraga CA (2007). Molecular hybridization: a useful tool in the design of new drug prototypes. Curr Med Chem.

[CR49] Wiegand I, Hilpert K, Hancock REW (2008). Agar and broth dilution methods to determine the minimal inhibitory concentration (MIC) of antimicrobial substances. Nat Protoc.

[CR50] Wood MR, Schirripa KM, Kim JJ, Wan B-L, Murphy KL, Random RW, Chang RSL, Tang C, Prueksaritanont T, Detwiler TJ, Hettrick LA, Landis ER, Leonard YM, Krueger JA, Lewis SD, Pettibone DJ, Freidinger RM, Bock MG (2006). Cyclopropylamino acid amide as a pharmacophoric replacement for 2,3-diaminopyridine. Application to the design of novel bradykinin B1 receptor antagonists. J Med Chem.

[CR51] Zaidi KU, Mani A, Parmar R, Thawan V (2018). Antifungal susceptibility pattern of *Candida albicans* in human infections. Open Biol Sci J.

[CR52] Zohra M, Fawzia A (2014). Hemolytic activity of different herbal extracts used in Algeria. Int J Pharm Sci Res.

