# Supplementary material for: The newly synthesized thiazole derivatives as potential antifungal compounds against Candida albicans
Source: Appl Microbiol Biotechnol. 2021 Aug 19;105(16-17):6355–67. doi: 10.1007/s00253-021-11477-7 (PMC8374424; doi:10.1007/s00253-021-11477-7)
Supplement: Supplementary file 1 — Supplementary file1 (PDF 358 kb) [file 253_2021_11477_MOESM1_ESM.pdf]

Applied Microbiology and Biotechnology

The newly synthesized thiazole derivatives as potential antifungal compounds against *Candida albicans*

Anna Biernasiuk[1]*, Anna Berecka-Rycerz[2], Anna Gumieniczek[2], Maria Malm[3], Krzysztof Z. Łączkowski[4], Jolanta Szymańska[5], Anna Malm[1]

[1] Department of Pharmaceutical Microbiology, Faculty of Pharmacy, Medical University of Lublin, Chodźki 1, 20-093 Lublin, Poland; anna.biernasiuk@umlub.pl (AB); anna.malm@umlub.pl (AM)
[2] Department of Medicinal Chemistry, Faculty of Pharmacy, Medical University of Lublin, Jaczewskiego 4, 20-090 Lublin, Poland; anna.berecka@umlub.pl (ABR); anna.gumieniczek@umlub.pl (AG)
[3] Department of Medicinal Informatics and Statistics with E-learning Lab, Faculty of Health Sciences, Medical University of Lublin, Jaczewskiego 4, 20-090 Lublin, Poland; maria.malm@umlub.pl (MM)
[4] Department of Chemical Technology and Pharmaceuticals, Faculty of Pharmacy, Collegium Medicum, Nicolaus Copernicus University, Jurasza 2, 85-089 Bydgoszcz, Poland; krzysztof.laczkowski@cm.umk.pl (KZL)
[5] Department of Integrated Paediatric Dentistry, Chair of Integrated Dentistry, Faculty of Medical Dentistry, Medical University of Lublin, Lubartowska 58, 20-94 Lublin, Poland; jolanta.szymanska@umlub.pl (JS)

* Correspondence: anna.biernasiuk@umlub.pl; ID ORCID: 0000-0002-2844-9876; Tel.: +48 81448 7100 (AB)

(a)

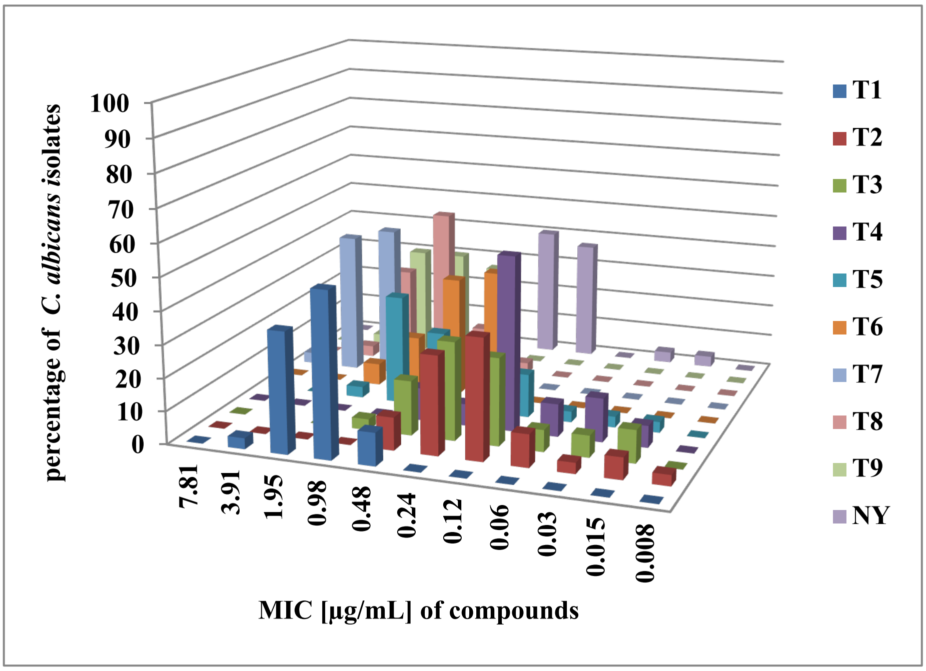

(b)

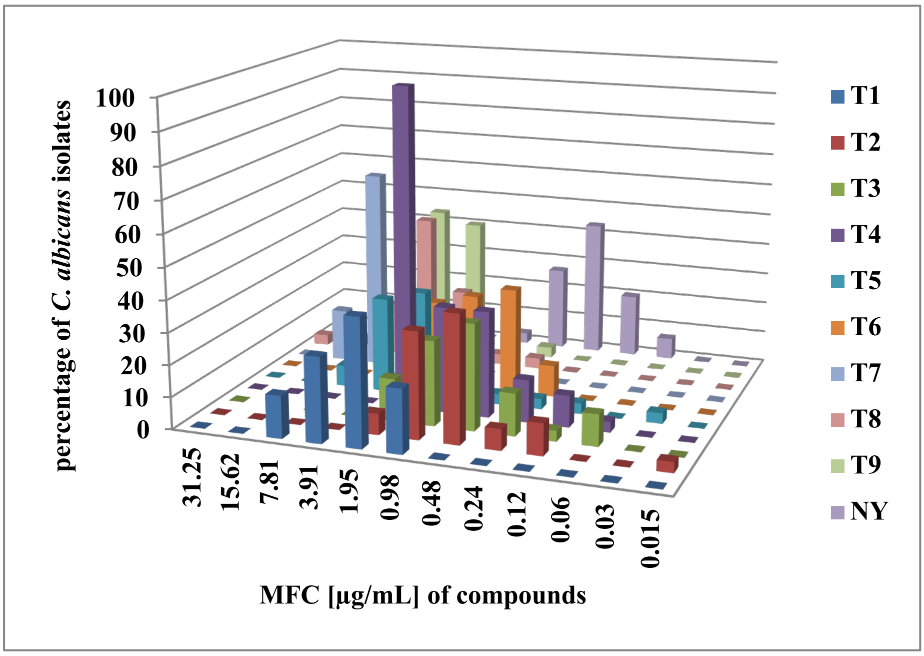

Figure S1 Distribution of: (a) MIC and (b) MFC values [µg/mL] of the newly synthesized thiazole derivatives among 30 clinical isolates of *C. albicans* from hospitalized patients with hematological malignancies. The standard antibiotic – nystatin (NY) was used as positive control

Table S1 MICs and FIC indexes of the newly synthesized thiazole derivatives alone and in combination with nystatin (NY) on reference *C. albicans* strains

***C. albicans* strains**	**Antifungal agent**	**MIC of antifungal agent (µg/mL)**		**FIC**	**Σ FIC**	**Interpretation**
		**Alone**	**Combination**			
C. albicans ATCC 10231	T1	1.95	1.95	1	2	Indifference
	NY	0.48	0.48	1		
C. albicans ATCC 2091	T1	1.95	1.95	1	3	Indifference
	NY	0.24	0.48	2		
C. albicans ATCC 10231	T2	0.06	0.06	1	3	Indifference
	NY	0.48	0.98	2		
C. albicans ATCC 2091	T2	0.12	0.24	2	3	Indifference
	NY	0.24	0.24	1		
C. albicans ATCC 10231	T3	0.12	0.24	2	4	Indifference
	NY	0.48	0.98	2		
C. albicans ATCC 2091	T3	0.12	0.24	2	3	Indifference
	NY	0.24	0.24	1		
C. albicans ATCC 10231	T4	0.06	0.12	2	4	Indifference
	NY	0.48	0.98	2		
C. albicans ATCC 2091	T4	0.12	0.24	2	4	Indifference
	NY	0.24	0.48	2		
C. albicans ATCC 10231	T5	0.015	0.015	1	3	Indifference
	NY	0.48	0.98	2		
C. albicans ATCC 2091	T5	0.24	0.24	1	2	Indifference
	NY	0.24	0.24	1		
C. albicans ATCC 10231	T6	0.48	0.48	1	2	Indifference
	NY	0.48	0.48	1		
C. albicans ATCC 2091	T6	1.95	1.95	1	3	Indifference
	NY	0.24	0.48	2		
C. albicans ATCC 10231	T7	0.48	0.98	2	4	Indifference
	NY	0.48	0.98	2		
C. albicans ATCC 2091	T7	1.95	1.95	1	3	Indifference
	NY	0.24	0.48	2		
C. albicans ATCC 10231	T8	0.24	0.48	2	4	Indifference
	NY	0.48	0.98	2		
C. albicans ATCC 2091	T8	0.98	0.98	1	3	Indifference
	NY	0.24	0.48	2		
C. albicans ATCC 10231	T9	0.48	0.48	1	2	Indifference
	NY	0.48	0.48	1		
C. albicans ATCC 2091	T9	3.91	3.91	1	3	Indifference
	NY	0.24	0.48	2		

Table S2 MICs and FIC indexes of the newly synthesized thiazole derivatives alone and in combination with chlorhexidine (CHX) on reference *C. albicans* strains

***C. albicans* strains**	**Antifungal agent**	**MIC of antifungal agent (µg/mL)**		**FIC**	**Σ FIC**	**Interpretation**
		**Alone**	**Combination**			
C. albicans	T1	1.95	1.95	1	2	Indifference
ATCC 10231	CHX	3.91	3.91	1		
C. albicans	T1	1.95	1.95	1	2	Indifference
ATCC 2091	CHX	1.95	1.95	1		
C. albicans	T2	0.06	0.06	1	2	Indifference
ATCC 10231	CHX	3.91	3.91	1		
C. albicans	T2	0.12	0.24	2	3	Indifference
ATCC 2091	CHX	1.95	1.95	1		
C. albicans	T3	0.12	0.12	1	2	Indifference
ATCC 10231	CHX	3.91	3.91	1		
C. albicans	T3	0.12	0.24	2	3	Indifference
ATCC 2091	CHX	1.95	1.95	1		
C. albicans	T4	0.06	0.06	1	2	Indifference
ATCC 10231	CHX	3.91	3.91	1		
C. albicans	T4	0.12	0.24	2	3	Indifference
ATCC 2091	CHX	1.95	1.95	1		
C. albicans	T5	0.015	0.015	1	2	Indifference
ATCC 10231	CHX	3.91	3.91	1		
C. albicans	T5	0.24	0.24	1	2	Indifference
ATCC 2091	CHX	1.95	1.95	1		
C. albicans	T6	0.48	0.98	2	3	Indifference
ATCC 10231	CHX	3.91	3.91	1		
C. albicans	T6	1.95	1.95	1	2	Indifference
ATCC 2091	CHX	1.95	1.95	1		
C. albicans	T7	0.48	0.48	1	2	Indifference
ATCC 10231	CHX	3.91	3.91	1		
C. albicans	T7	1.95	1.95	1	2	Indifference
ATCC 2091	CHX	1.95	1.95	1		
C. albicans	T8	0.24	0.24	1	2	Indifference
ATCC 10231	CHX	3.91	3.91	1		
C. albicans	T8	0.98	0.98	1	2	Indifference
ATCC 2091	CHX	1.95	1.95	1		
C. albicans	T9	0.48	0.98	2	3	Indifference
ATCC 10231	CHX	3.91	3.91	1		
C. albicans	T9	3.91	3.91	1	2	Indifference
ATCC 2091	CHX	1.95	1.95	1		

Table S3 MICs and FIC indexes of the newly synthesized thiazole derivatives alone and in combination with thymol (THY) on reference *C. albicans* strains

***C. albicans* strains**	**Antifungal agent**	**MIC of antifungal agent (µg/mL)**		**FIC**	**Σ FIC**	**Interpretation**
		**Alone**	**Combination**			
C. albicans	T1	1.95	1.95	1	1.5	Indifference
ATCC 10231	THY	125	62.5	0.5		
C. albicans	T1	1.95	1.95	1	1.25	Indifference
ATCC 2091	THY	250	62.5	0.25		
C. albicans	T2	0.06	0.03	0.5	1	Additivity
ATCC 10231	THY	125	62.5	0.5		
C. albicans	T2	0.12	0.06	0.5	1	Additivity
ATCC 2091	THY	250	125	0.5		
C. albicans	T3	0.12	0.06	0.5	1	Additivity
ATCC 10231	THY	125	62.5	0.5		
C. albicans	T3	0.12	0.12	1	1.25	Indifference
ATCC 2091	THY	250	62.5	0.25		
C. albicans	T4	0.06	0.06	1	1.5	Indifference
ATCC 10231	THY	125	62.5	0.5		
C. albicans	T4	0.12	0.12	1	1.25	Indifference
ATCC 2091	THY	250	62.5	0.25		
C. albicans	T5	0.015	0.0075	0.5	1	Additivity
ATCC 10231	THY	125	62.5	0.5		
C. albicans	T5	0.24	0.12	0.5	1	Additivity
ATCC 2091	THY	250	125	0.5		
C. albicans	T6	0.48	0.48	1	1.5	Indifference
ATCC 10231	THY	125	62.5	0.5		
C. albicans	T6	1.95	1.95	1	1.5	Indifference
ATCC 2091	THY	250	125	0.5		
C. albicans	T7	0.48	0.24	0.5	1	Additivity
ATCC 10231	THY	125	62.5	0.5		
C. albicans	T7	1.95	1.95	1	1.25	Indifference
ATCC 2091	THY	250	62.5	0.25		
C. albicans	T8	0.24	0.24	1	1.5	Indifference
ATCC 10231	THY	125	62.5	0.5		
C. albicans	T8	0.98	0.98	1	1.25	Indifference
ATCC 2091	THY	250	62.5	0.25		
C. albicans	T9	0.48	0.98	2	2.5	Indifference
ATCC 10231	THY	125	62.5	0.5		
C. albicans	T9	3.91	3.91	1	2	Indifference
ATCC 2091	THY	250	250	1		
